# Corrigendum

**DOI:** 10.1111/iwj.13841

**Published:** 2022-07-15

In the article [1], there is an error in Table 5 on page 939 which also affected a sentence on page 941 under section 3 Results, second sentence in sixth paragraph.

**TABLE 5:** Logistic regression, dependent variable of healed or not, and independent variables as listed

Variable	B	*p*	OR	95% CI
BSA^1^	1.91	**0.00015**	6.7	2.52–18.02
Ethnicity^2^	0.007	0.92	1.08	0.28–4.14
African American Other^a^	1.72	**0.045**	5.57	1.04–29.83
Area at randomization (cm^2^)	−0.13	**0.014**	0.88	0.79–0.97
BMI	−0.071	0.094	0.93	0.86–1.01
Constant	3.54	**0.017**	34.33	

Note: Reference groups: ^1^SOC; ^2^Caucasian. Bold values are statistically significant (*P* < .05).

Abbreviations: CI, confidence interval; OR, odds ratio.

^a^ Includes Hispanic, Asian, Middle‐Eastern, and Native Hawaiian races/ethnicities.

The OR value 6.74 for BSA^1^ was incorrect in Table 5. The correct value should have been 6.7. The correct data is shown below:

Correspondingly, the following sentence on page 941,

The treatment (BSA) dominates the other variables and increases the odds of healing by 6.74 (P = .00015).

Should have read:

The treatment (BSA) dominates the other variables and increases the odds of healing by 6.7 (P = .00015).

## References

[1] Armstrong DG , Galiano RD , Orgill DP , et al. Multi‐centre prospective randomised controlled clinical trial to evaluate a bioactive split thickness skin allograft vs standard of care in the treatment of diabetic foot ulcers. Int Wound J. 2022;19(4):932‐944. doi:10.1111/iwj.13759 35080127 PMC9013597

